# Successful Collection of Patient-Reported Outcomes Shows Improvement in Quality of Life, Depression, and Disease Activity among Patients with Inflammatory Bowel Disease: A Real-World Study

**DOI:** 10.1093/crocol/otaf064

**Published:** 2025-11-13

**Authors:** Cihang Gu, Audrey Bennett, Justin Bachmann, David A Schwartz, Dawn Beaulieu, Elizabeth Scoville, Robin Dalal, Baldeep Pabla, Allison McCoy, James C Slaughter, Sara Horst

**Affiliations:** Division of General Internal Medicine, Department of Medicine, University of Pittsburgh Medical Center, Pittsburgh, PA, United States; Division of Gastroenterology, Hepatology and Nutrition, Department of Medicine, Vanderbilt University Medical Center, Nashville, TN, United States; Division of Cardiovascular Medicine, Department of Medicine, Vanderbilt University Medical Center, Nashville, TN, United States; Division of Gastroenterology, Hepatology and Nutrition, Department of Medicine, Vanderbilt University Medical Center, Nashville, TN, United States; Division of Gastroenterology, Hepatology and Nutrition, Department of Medicine, Vanderbilt University Medical Center, Nashville, TN, United States; Division of Gastroenterology, Hepatology and Nutrition, Department of Medicine, Vanderbilt University Medical Center, Nashville, TN, United States; Division of Gastroenterology, Hepatology and Nutrition, Department of Medicine, Vanderbilt University Medical Center, Nashville, TN, United States; Division of Gastroenterology, Hepatology and Nutrition, Department of Medicine, Vanderbilt University Medical Center, Nashville, TN, United States; Department of Biomedical Informatics, Vanderbilt University Medical Center, Nashville, TN, United States; Department of Biostatistics, Vanderbilt University Medical Center, Nashville, TN,United States; Division of Gastroenterology, Hepatology and Nutrition, Department of Medicine, Vanderbilt University Medical Center, Nashville, TN, United States

**Keywords:** patient-reported outcomes, quality of life, depression, disease activity, Crohn’s disease, ulcerative colitis

## Abstract

**Introduction:**

Patient-reported outcomes (PRO), including patient disease activity scores, quality of life, and depressive symptoms, are increasingly being used for clinical care in patients with inflammatory bowel disease (IBD). However, little is known about the performance of PROs over time in a real-world setting.

**Methods:**

PROs were collected from a tertiary care IBD center from the electronic medical records (EMR) from 2018 to 2020. Quality of life was measured with the Simple Inflammatory Bowel Disease Questionnaire (SIBDQ). Disease activity was measured using the Harvey Bradshaw Index (HBI) for Crohn’s disease (CD) and the Simple Clinical Colitis Activity Index (SCCAI) for ulcerative colitis (UC). Depressive symptoms were measured using the Patient Health Questionnaire-8 (PHQ-8).

**Results:**

PRO collection rate from 1373 patients was 88%. PHQ and SIBDQ were highly correlated (.718, *p* [Pearson’s coefficient] < .05). HBI and PHQ-8 (.528, *P* < .05) and SIBDQ (–.676, *P* < .05) were moderately correlated. For CD patients, age 40-65, corticosteroid use and psychiatric medication use were associated with lower HBI and SIBDQ and higher PHQ scores. Age 40-65, corticosteroid use, and psychiatric medication use were associated with lower SCCAI and SIBDQ and higher PHQ-8 scores for CD and UC patients. Mean change [95% CI] increased for SIBDQ by 2.9 [2.3 to 3.6] in CD and 4.7 [3.8 to 5.6] in UC. Mean change [95% CI] decreased for PHQ-8: −1.0 [−1.3 to −.8] in CD and −1.7 [−2.2 to −1.3] in UC. This remained significant in both cognitive and somatic subscales.

**Conclusion:**

High rate of PRO collection was successful using EMR implementation in a tertiary care clinic setting. Corticosteroid use and psychiatric medication use were associated with worse disease activity, depressive symptoms and quality of life scores. Moreover, PROs demonstrated depressive symptoms (both cognitive and somatic subscales), and quality of life symptom scores improved over time.

## Graphical abstract

**Graphical Abstract:**
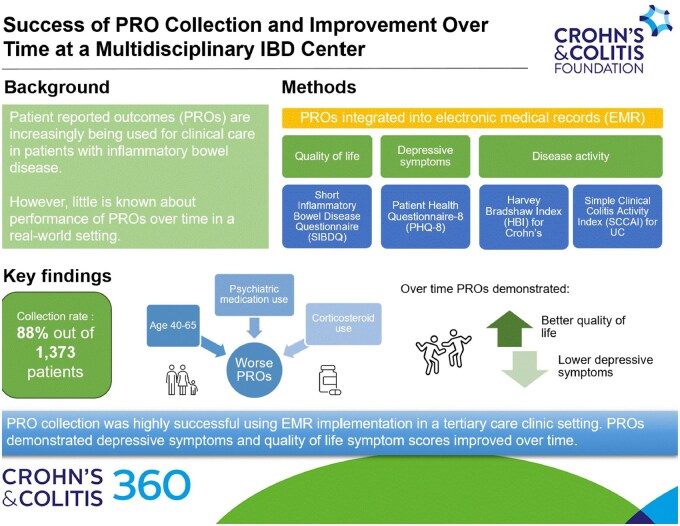

## Introduction

Crohn’s disease (CD) and ulcerative colitis (UC) are chronic inflammatory conditions of the gastrointestinal (GI) tract. When active, symptoms of inflammatory bowel disease (IBD) can include abdominal pain, diarrhea, rectal bleeding, bowel urgency, fatigue, and weight loss. In addition to physical symptoms, these chronic illnesses also have a profound impact on mental health and overall quality of life, affecting daily activities, school performance, work ability, and social life.^1-3^ While IBD management is typically focused on laboratory, radiologic, and endoscopic results, the assessment of success in IBD treatment has been evolving to include a holistic view of patients’ well-being.

One method of gaining insight into patient symptoms and quality of life is the use of patient-reported outcome (PRO) surveys. PROs allow for a quantifiable measure of patients’ perspectives on their health. Prior research on chronic disease has shown that patients and providers may have different perspectives on outcomes and that patients have additional treatment priorities.^4-7^ PROs are increasingly used in phased clinical trials to understand all aspects of patients’ disease experience, including disease activity measures, mental health comorbidities, and quality of life. Also, increasingly, clinical practice recommendations include monitoring improvement in patient-reported disease symptom measures as an important treatment target for patients with IBD.^8^ There is some small data across chronic disease states that monitoring PROs help clinicians track patients’ well-being, bridge gaps in perceived disease severity, and improve shared decision-making.^5^^,^^9^ Despite validated PROs developed for patients with IBD to monitor disease activity, quality of life, and depressive symptoms, little is known about the success of monitoring of PROs in a real-world clinical practice over time. Over the past decades, there has been a rapid growth in the capability of the electronic medical record (EMR) to more easily capture and follow PROs in usual clinical care settings.

The primary aim of this study was to assess how patient characteristics and disease phenotypes influence PRO scores for patients presenting to a multidisciplinary IBD clinic and evaluate the change in PROs over time.

## Methods

This retrospective study was conducted at a tertiary care, multidisciplinary IBD center and included adult patients with CD or UC. Data were collected as part of clinical care on an automated basis from the EMR from 2018 to 2020. The study ended in 2020 as the COVID-19 pandemic impacted workflow and data collection with a shift to telemedicine. Before each clinic visit, patients were instructed to complete surveys to assess quality of life, depressive symptoms, and disease activity. Patients had the option to complete the PRO surveys up to 72 hours before the clinic visit within the EMR patient portal on a home computer or mobile device as part of their pre–clinic check-in. Patients who had not completed the questionnaires before the clinic visit were asked to complete them prior to their clinic visit via tablets by the front desk staff at check-in and before the intake process. The results of the questionnaires for current and previous encounters were easily viewable in several areas of the EMR at the time of a clinic visit and auto-populated into the clinician’s note templates.

Quality of life was measured using the Short Inflammatory Bowel Disease Questionnaire (SIBDQ). Depression was measured using the Patient Health Questionnaire-8 (PHQ-8). Disease activity was measured using the Harvey Bradshaw Index (HBI) and the Simple Clinical Colitis Activity Index (SCCAI) for CD and UC, respectively. Detailed descriptions of the tools can be found in Supplementary Appendix 1.

Patients were included in this study if they were new patients to the IBD clinic at the start of the study in 2018. In addition to PRO survey data, patient charts were also reviewed to collect data including patient age, sex, ethnicity, insurance status, smoking status, IBD phenotype, prior IBD-related surgeries, and baseline medication use including psychiatric medications (selective serotonin reuptake inhibitor [SSRI]/serotonin and norepinephrine reuptake inhibitor [SNRI], atypical anti–depressant, benzodiazepine, and/or anti–psychotic medication), steroids, immunomodulators (methotrexate, 6-MP, and/or azathioprine), biologics (anti–tumor necrosis factor therapies, anti–integrin therapies, or anti–IL12/23 therapies), or small molecules (JAK inhibitors). Continuous nonparametric data is presented with median and range. Statistical analysis included the Pearson test to compare categorical data and the Wilcoxon rank test to compare continuous data. Analysis for PRO over time included a linear mixed model fit by REML with t-tests using Satterthwaite’s method.

### Ethical considerations

Institutional review board (IRB) approval at Vanderbilt University Medical Center was obtained for this retrospective chart review.

## Results

The overall PRO collection rate from 1373 patients was 88%. Of the patients in this study, 28.5% completed the PRO surveys via the patient portal before their visit, and 71.5% completed PRO surveys on a provided tablet the day of their clinic visit. Follow-up PRO data were available in 1139 subjects (82%). The median patient age was 36 (75% interquartile range of 25-52), the majority (87%) were white, and 43% were male. In this cohort, 66% had CD and 34% had UC, with further disease phenotypes outlined in Table 1. Of the patients in this study, 30% had prior surgery for their IBD. At the time of their first patient visit to the IBD center, 41% were using corticosteroids, 45% were on a biologic medication, 23% were on an immunomodulator, and 32% were on a psychiatric medication. Median baseline PRO scores included: SIBDQ 50 (range 39-59), PHQ-8 5 (range 2-11), SCCAI 5 (range 2-7), and HBI 5 (range 2-8).

**Table 1.** Baseline characteristics and patient reported outcomes.

	Patients *n* = 1376
**Age, median (range)**	36 (25, 52)
**Sex (male), % (*n*)**	43 (589)
**Crohn’s disease, % (*n*)**	66 (910)
**Race, % (*n*)**	
** White**	87 (1197)
** Black**	7 (93)
** Other**	6 (83)
**Smoker, % (*n*)**	
** Former**	23 (107)
** Current**	15 (201)
** None**	62 (845)
**Surgery, % (*n*)**	30 (417)
**Corticosteroid use, % (*n*)**	41 (564)
**Biologic, % (*n*)**	45 (617)
**Immunomodulator, % (*n*)**	23 (316)
**Psychiatric medication, % (*n*)**	32 (440)
**Crohn’s disease, % (*n*)**	
** Fistulizing**	7 (64)
** Perianal**	16 (147)
** Stricturing**	29 (262)
** Penetrating**	14 (123)
**Ulcerative colitis, % (*n*)**	
** Extensive**	71 (331)
** Left sided**	25 (115)
** Proctitis**	4 (17)
**Patient reported outcomes**	
** SIBDQ, median (range)**	50 (37, 60)
** PHQ 8, median (range)**	5 (2, 11)
** SCCAI (UC), median (range)**	5 (2, 7)
** HBI (CD), median (range)**	5 (2, 8)

Among both UC and CD patients, depressive symptoms (PHQ-8) and quality of life (SIBDQ) were highly inversely correlated with a Spearman correlation of −.757, *P* < .05. For CD patients, disease activity (HBI) and depressive symptoms (PHQ-8) were positively correlated with a Spearman correlation of .528, *P* < .05, and disease activity (HBI) and quality of life (SIBDQ) were moderately inversely correlated with a Spearman correlation of −.673, *P* < .05. For UC, disease activity (SCCAI) and depressive symptoms (PHQ-8) were positively correlated with a Spearman correlation of .540, *P* < .05, and disease activity (SCCAI) and quality of life (SIBDQ) were moderately inversely correlated with a Spearman correlation of −.789, *P* < .05.

In patients with CD, age 40-65, baseline corticosteroid use, and psychiatric medication use were associated with higher patient-reported disease activity (HBI), higher depressive symptoms (PHQ-8), and lower quality of life (SIBDQ) at baseline (Table 2). For patients with UC, baseline corticosteroid use was associated with higher patient-reported disease activity (SCCAI), higher depressive symptoms (PHQ-8), and decreased quality of life (SIBDQ) at baseline. UC patients taking psychiatric medications at baseline also reported higher depressive symptom scores (PHQ-8) and decreased quality of life (SIBDQ) (Table 2).

**Table 2.** Univariate analysis of patient reported outcomes at baseline.

**Crohn’s disease (*n*** **=** **910)**		HBI	*P*	PHQ 8	*P*	SIBDQ	*P*
**Age range**	<40	4 (1, 7)	<.001	5 (2, 10)	<.001	52 (39, 62)	<.001
	40-65	6 (3, 10)		7 (2, 12)		46 (33, 57)	
	>65	4 (1, 7)		3 (1, 8)		53 (43, 60)	
**Corticosteroid use**	Yes	6 (4, 9)	<.001	7 (3, 11)	<.001	46 (34, 55)	<.001
	No	4 (1, 7)		5 (2, 11)		54 (40, 62)	
**Biologic use**	Yes	5 (2, 7)	NS	5 (2, 11)	NS	51 (39, 60)	NS
	No	5 (2, 8)		6 (2, 11)		50 (37, 60)	
**IMM use**	Yes	5 (2, 9)	NS	5 (2, 11)	NS	51 (37, 60)	NS
	No	5 (2, 9)		6 (2, 11)		50 (39, 58)	
**Psychiatric med use**	Yes	6 (4, 10)	<.001	9 (4, 13)	<.001	42 (31, 55)	<.001
	No	4 (1, 9)		4 (1, 9)		54 (41, 62)	

UC (*n* = 463)		SSCAI		PHQ 8		SIBDQ	
**Age range**	<40	5 (2, 8)	NS	6 (2, 11)	NS	46 (35, 60)	NS
	40-65	5 (2, 7)		5 (2, 9)		49 (39, 60)	
	>65	5.5 (3.8, 8)		6.5 (1.5, 10)		43 (33, 57)	
**Corticosteroid use**	Yes	6 (4, 9)	<.001	7 (3, 11)	<.001	42 (33, 55)	<.001
	No	4 (1, 7)		5 (2, 11)		51 (41, 63)	
**Biologic use**	Yes	5 (2, 8)	NS	6 (2, 11)	NS	46 (36, 59)	NS
	No	5 (2, 8)		5 (2, 8)		48 (38, 60)	
**IMM use**	Yes	5 (2, 8)	NS	8 (3, 12)	0.05	44 (33, 55)	NS
	No	6 (4, 8)		5 (2, 10)		48 (38, 60)	
**Psychiatric med use**	Yes	6 (3, 7)	NS	7 (4, 12)	<.001	44 (33, 53)	<.001
	No	4 (2, 8)		5 (1, 10)		48 (39, 62)	

**Figure 1.**
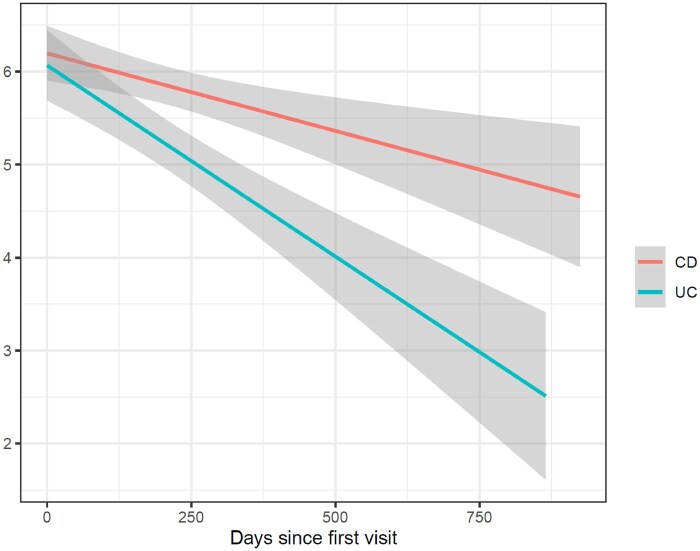
PHQ 8 significantly decreases over time in patients with inflammatory bowel disease.

**Figure 2.**
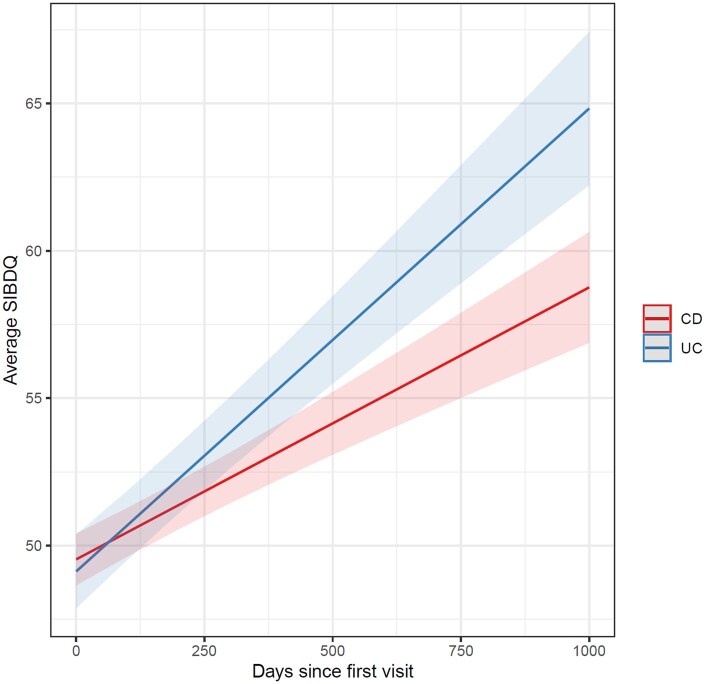
SIBDQ significantly increases over time in patients with inflammatory bowel disease.

The majority of patients had a return visit to the IBD clinic (*n* = 881, 70%), and 50% (*n* = 620) returned for at least 3 visits. Depressive symptoms (PHQ-8) and quality of life (SIBDQ) improved significantly over time for UC and CD patients (­Figures 1 and 2). The mean SIBDQ [95% CI] significantly increased in patients with CD by 3.4 per year [2.6 to 4.1] and in patients with UC by 5.7 per year [4.7 to 4.8]. The mean PHQ-8 [95% CI] significantly decreased in patients with CD by −1.0 per year [−1.3 to −.8] and in patients with UC by −1.7 per year [−2.2 to −1.3]. This remained significant in PHQ-8 somatic subscale decrease by −.7 per year [−.9 to −.5] and PHQ-8 cognitive subscale by −.4 per year [−.5 to −.2] in patients with CD and for PHQ-8 somatic subscale by −1.1 per year [−1.3 to −.8] and PHQ-8 cognitive subscale by −.7 per year [−.9 to −.5]) in patients with UC.

In evaluation of specific disease characteristics (age, sex, and for CD: perianal, penetrating, or stricturing disease) and medication use (corticosteroid use, biologic use, immunomodulator use, psychiatric medication use), significant improvement for all PROs persisted in CD and UC for patients who had follow up evaluation with statistically significant differences. Importantly, slopes and confidence intervals between groups were overlapping in all subgroups, indicating no significant difference in rate of change over time between groups (Table 3). The only subtypes that did not reach statistical significance for PHQ-8 improvement included patients with age >65 for UC and CD and patients with perianal CD. For quality of life improvement, the only patient scores that did not reach statistical significance were patients with CD who were >age 65, had perianal disease or fistulizing disease.

**Table 3.** Time effect of PRO changes comparing medication use and disease type in patients with ulcerative colitis and Crohn’s disease.

Ulcerative colitis		SIBDQ (Mean change [95% CI])	PHQ-8 (Mean change [95% CI])	SCCAI (Mean change [95% CI])
**Sex**				
	Male	4.9 [3.6 to 6.2]	−1.8 [−2.4 to −1.3]	−1.5 [−1.9 to −1.1]
	Female	4.7 [3.3 to 6.1]	−1.7 [−2.3 to −1.0]	−1.5 [−1.9 to −1.0]
**Corticosteroid**				
	No	3.6 [2.1 to 5.0]	−1.0 [−1.6 to −0.4]	−1.0 [−1.5 to −0.6]
	Yes	5.9 [4.6 to 7.2]	−2.4 [−2.9 to −1.8]	−1.9 [−2.3 to −1.5]
**Biologic**				
	No	4.8 [3.5 to 6.0]	−1.5 [−2.0 to v0.9]	−1.4 [−1.8 to −1.0]
	Yes	4.9 [3.4 to 6.4]	−2.2 [−2.8 to −1.5]	−1.6 [−2.1 to −1.1]
**IMM**				
	No	4.6 [3.5 to 5.8]	−1.6 [−2.1 to −1.1]	−1.3 [−1.7 to −1.0]
	Yes	5.1 [3.3 to 6.8]	−2.2 [−2.9 to −1.4]	−1.7 [−2.3 to −1.2]
**Psychiatric med**				
	No	5.7 [4.5 to 6.9]	−2.0 [−2.5 to −1.4]	−1.6 [−2.0 to −1.2]
	Yes	3.8 [2.3 to 5.4]	−1.6 [−2.2 to −0.9]	−1.3 [−1.8 to −0.8]
**Age**				
	<40	4.8 [3.5 to 6.1]	−1.8 [−2.4 to −1.3]	−1.3 [−1.7 to −0.9]
	40-65	5.2 [3.5 to 6.9]	−1.9 [−2.7 to −1.2]	−1.6 [−2.2 to −1.0]
	>65	3.7 [1.2 to 6.3]	−1.1 [−2.2 to 0.0]^**^	−1.7 [−2.5 to −0.9]

Crohn’s disease		SIBDQ (Mean change [95% CI])	PHQ-8 (Mean change [95% CI])	HBI (Mean change [95% CI])
**Sex**				
	Male	3.0 [2.2 to 3.8]	−1.3 [−1.7 to −1.0]	−0.8 [−1.2 to −0.5]
	Female	2.8 [1.8 to 3.8]	−0.6 [−1.1 to −0.2]	−0.4 [−0.8 to 0.0]
**Corticosteroid**				
	No	3.0 [2.1 to 3.9]	−1.2 [−1.6 to −0.8]	−0.5 [−0.9 to −0.2]
	Yes	3.0 [2.1 to 3.9]	−1.0 [−1.4 to −0.5]	−0.8 [−1.2 to −0.5]
**Biologic**				
	No	3.6 [2.4 to 4.8]	−1.3 [−1.9 to −0.8]	−1.0 [−1.5 to −0.5]
	Yes	2.7 [2.0 to 3.4]	−1.0 [−1.3 to −0.6]	−0.5 [−0.8 to −0.2]
**IMM**				
	No	2.9 [2.1 to 3.6]	−1.0 [−1.4 to −0.6]	−0.6 [−0.9 to −0.3]
	Yes	3.1 [2.1 to 4.1]	−1.2 [−1.6 to −0.7]	−0.8 [−1.2 to −0.3]
**Psychiatric med**				
	No	3.5 [2.7 to 4.3]	−0.9 [−1.3 to v0.6]	−0.7 [−1.1 to −0.4]
	Yes	2.4 [1.4 to 3.3]	−1.4 [−1.9 to −0.9]	−0.6 [−1.0 to −0.2]
**Age**				
	<40	2.8 [2.0 to 3.6]	−1.0 [−1.3 to −0.6]	−0.5 [−0.8 to −0.2]
	40-65	3.0 [2.0 to 4.1]	−1.3 [−1.8 to −0.8]	−0.9 [−1.4 to −0.5]
	>65	3.5 [0.7 to 6.3]	−0.8 [−2.1 to 0.5]^**^	−0.9 [−2.1 to 0.3]^**^
**Perianal disease**				
	No	3.2 [2.4 to 3.9]	−1.1 [−1.4 to −0.8]^**^	−0.6 [−1.0 to −0.3]^**^
	Yes	2.5 [1.0 to 4.0]	−0.5 [−1.2 to 0.2]	−0.6 [−1.3 to 0.0]
**Stricturing disease**				
	No	2.9 [2.2 to 3.6]	−1.1 [−1.4 to −0.7]	−0.6 [−0.9 to −0.3]
	Yes	3.2 [1.9 to 4.5]	−1.0 [−1.6 to −0.3]	−0.8 [−1.3 to −0.2]
**Fistulizing disease**				
	No	2.9 [2.2 to 3.5]	−1.0 [−1.3 to −0.7]	−0.7 [−1.0 to −0.4]
	Yes	3.3 [1.5 to 5.1]	−1.6 [−2.5 to −0.8]	−0.3 [−1.1 to 0.4]^**^

** If statistically significant.

## Discussion

This study demonstrates that PRO collection over time was highly successful using EMR collection in a real-world setting. Other studies have shown PRO collection at lower rates with differing methods of collection outside of the EMR.^10^ In our study, patients with certain characteristics at a new visit, such as age range 40-65 years old, corticosteroid use, or psychiatric medication use, had significantly lower PROs. While characteristics such as baseline corticosteroid use associated with higher disease activity may be expected, it is important to see this correlation with other PROs in a real-world clinical setting. This affirms that these easily obtainable clinical characteristics can be evaluated to understand those at risk for lower PROs. Additionally, this study found that PROs improve over time at a tertiary referral multidisciplinary IBD center regardless of individual patient and disease characteristics. Prior studies have shown that digitally collected PROs can play a valuable role in remote monitoring and shared medical decision-making.^11^^,^^12^ This study suggests that EMR integration of PRO capture is a feasible and practical tool in clinical care of IBD patients and beneficial to assessing and monitoring patient outcomes. After initial integration, PRO collection does not require additional personnel or clinical time. This creates a wealth of additional clinical data that can be used to track patient outcomes and used in research with ease. As an institutional effort to promote integration of PROs into the EMR, other clinics have also had success with PRO collection in a similar fashion, suggesting that this model of PRO collection can be generalized.^13^

PROs are becoming a standard measure of IBD symptoms in terms of therapeutic targets and clinical trial endpoints, spanning domains such as quality of life, fatigue, work productivity, depression, and anxiety.^8^^,^^14^ PRO2, which includes the daily stool frequency and abdominal pain items from the Crohn’s Disease Activity Index, has been validated to assess clinical remission^15^ and has been shown to be easily collected via remote monitoring from patients in the real world.^16^ Interestingly, studies have suggested mild discrepancies between endoscopic healing and symptom-based PROs in IBD patients,^17^ which may help identify additional gaps in patients’ care beyond objective assessments of disease activity. Patient-reported pain has been associated with poor psychosocial outcomes and poor quality of life, as well as a higher risk of hospitalization.^18^^,^^19^ PROs can also be helpful tools in triaging outpatient care needs, can serve as a proxy for monitoring, and reduce the number of visits.^20^

In addition to disease activity, PROs may also help assess the impacts of chronic disease on health-related quality of life. Psychological symptoms can have a complex interplay with chronic disease as well, and may be important to evaluate. Depression and anxiety have a bidirectional relationship with IBD. Depression is a modifiable risk factor that is associated with higher hospitalization rates, readmission rates, therapy nonadherence, disability rates, and rates of clinical recurrence in patients with IBD.^21–24^ Treating depression has been shown to be beneficial for IBD activity and lower rates of treatment escalation.^25^^,^^26^ Therefore, identifying those who are at risk for depression and anxiety is crucial to the overarching treatment of IBD. Screening for anxiety and depression is increasingly being recommended in clinical guidelines.^27^ Using PROs to screen for psychiatric comorbidities can help stratify IBD patients and identify those with potential depression.^28^ This identification can help allow early intervention to address, treat, and manage these psychiatric comorbidities.

Importantly, these PROs can be highly interrelated. For example, depression and anxiety has been shown to be associated with worse quality of life, even independent of objective symptoms.^29^^,^^30^ Treatment of depression through antidepressants and psychotherapy is associated with improved quality of life.^31^^,^^32^ In our study, quality of life scores and depressive symptoms were very highly correlated. This suggests that evaluation for either quality of life or depressive symptoms may be adequate. These depressive symptoms and quality of life scores were also moderately correlated with disease activity scores. This is consistent with prior studies that have found the prevalence of depression and anxiety symptoms is higher in patients with active IBD compared to those in remission.^33^

Secondary analysis showed that baseline corticosteroid use or psychiatric medication use was associated with worse disease activity (HBI or SCCAI), depressive symptoms (PHQ-8), and quality of life scores (SIBDQ) and could be surrogate markers clinically for those at risk for worsened PROs. This study also showed that for patients who returned to the clinical setting, all PROs improved over time. This was regardless of patient or disease characteristics. This improvement may be due to prompt identification of disease activity or comorbidities and early triaging of proper care with PROs. Additionally, the multidisciplinary model of care with IBD specialists and ancillary providers readily available may have also benefited the patients. In this patient population, multi-disciplinary comprehensive care allowed for this improvement over time. This highlights the importance of comprehensive care in patients with IBD.

Limitations of this study include its retrospective design and the disruption of data collection due to the COVID-19 pandemic, with a sudden change to workflows. The follow-up PROs relied on patients returning to the clinical setting, limiting the patient population. This was a single-center retrospective study; however, this may have been beneficial in the evaluation of PROs over time to limit bias associated with differential care that would have occurred at different sites. Detailed follow-up changes to management including medications and psychological care, were not available in this current study, as well as the frequency and duration of follow-ups. This may be a point of interest for future studies to examine how clinicians reacted to changes in PROs over time that led to improved patient outcomes. Our study showed that PROs can be successfully integrated into clinical workflows with EMR standardization. Further investigation can help delineate the clinical implications of PRO collection and how these can impact management decisions.

## Supplementary Material

otaf064_Supplementary_Data

## Data Availability

Data are available by emailing the corresponding author.
