# Supplementary material for: Successful Collection of Patient-Reported Outcomes Shows Improvement in Quality of Life, Depression, and Disease Activity among Patients with Inflammatory Bowel Disease: A Real-World Study
Source: Crohns Colitis 360. 2025 Nov 13;7(4):otaf064. doi: 10.1093/crocol/otaf064 (PMC12687582; doi:10.1093/crocol/otaf064)
Supplement: otaf064_Supplementary_Data [file otaf064_supplementary_data.zip › Appendix 1.docx]

Appendix 1:

SIBDQ: The SIBDQ is a validated, 10-item questionnaire that evaluates four domains of quality of life: physical, social, emotional, and systemic. The SIBDQ is scored on a 7-point Likert scale from 1 (severe) to 7 (no problems). The score ranges from 10 (poor quality of life) to 70 (high quality of life).^34,35^

PHQ-8: This validated questionnaire evaluates a patient’s mental health over the prior two weeks. Answers for each of the eight questions range from ‘not at all’, ‘several days’, ‘more than half of the days’, and ‘nearly every day’, correlating with scores of 0 to 3, respectively. Overall, PHQ-8 scores range from 0-24. Prior studies have shown scores ≥10 are associated with depression.^36^

HBI: The HBI is a validated questionnaire created to be an abbreviated version of the Crohn’s Disease Activity Index (CDAI) survey. ^37,38^ This survey evaluates general well-being on a scale of 0 to 4, with 0 being ‘very well’ and 4 being ‘terrible’, abdominal pain on a scale of 0 to 3, with 0 being ‘none’ and 3 being ‘severe’, number of liquid or soft stools per day with one point per stool, abdominal mass from 0 to 3 with 0 being ‘none’ and 3 being ‘definite and tender’, as well as complications with 1 point each for arthralgias, uveitis, erythema nodosum, aphthous ulcers, pyoderma gangrenosum, anal fissures, new fistula, or abscess. Scores < 5 represent remission, 5-7 mild disease, 8-16 moderate disease, and > 16 severe disease.^39^

SCCAI: The SCCAI is a validated questionnaire that evaluates six variables including bowel frequency during the day, bowel frequency at night, bowel urgency, blood in stool, general well-being, and extraintestinal manifestations of UC.^40,41^ Total scores range from 0 to 19 with higher values representing worse disease and scores < 2.5 representing remission.^42^
